# Recurrence in Oral Premalignancy: Clinicopathologic and Immunohistochemical Analysis

**DOI:** 10.3390/diagnostics11050872

**Published:** 2021-05-12

**Authors:** Maria Georgaki, Dimitris Avgoustidis, Vasileios Ionas Theofilou, Evangelia Piperi, Efstathios Pettas, Demos G. Kalyvas, Dimitrios Vlachodimitropoulos, Christos Perisanidis, Andreas C. Lazaris, Nikolaos G. Nikitakis

**Affiliations:** 1 Department of Oral Medicine & Pathology and Hospital Dentistry, School of Dentistry, National and Kapodistrian University of Athens, 11527 Athens, Greece; vasilistheofilou@gmail.com (V.I.T.); liapiperi@dent.uoa.gr (E.P.); stathis.pettas12@gmail.com (E.P.); nnikitakis1@yahoo.com (N.G.N.); 2 Department of Oral and Maxillofacial Surgery, “Evaggelismos” General Hospital, School of Dentistry, National and Kapodistrian University of Athens, 10676 Athens, Greece; davgoustidis@gmail.com; 3 Department of Oncology and Diagnostic Sciences, School of Dentistry, University of Maryland, Baltimore, MD 21201, USA; 4 Department of Oral & Maxillofacial Surgery, School of Dentistry, National and Kapodistrian University of Athens, 11527 Athens, Greece; Demos.Kalyvas@dent.uoa.gr (D.G.K.); cperis@dent.uoa.gr (C.P.); 5 Department of Forensic Medicine-Toxicology, School of Medicine, National and Kapodistrian University of Athens, 11527 Athens, Greece; dvlacho@med.uoa.gr; 6 Department of Pathology, School of Medicine, National and Kapodistrian University of Athens, 11527 Athens, Greece; alazaris@med.uoa.gr

**Keywords:** oral leukoplakia, oral potentially malignant disorders, recurrence, laser ablation, cyclin D1, STAT3, Bcl-xL, survivin, Ki-67, predictive biomarkers

## Abstract

Oral leukoplakia (OL) has a propensity for recurrence and malignant transformation (MT). Herein, we evaluate sociodemographic, clinical, microscopic and immunohistochemical parameters as predictive factors for OL recurrence, also comparing primary lesions (PLs) with recurrences. Thirty-three patients with OL, completely removed either by excisional biopsy or by laser ablation following incisional biopsy, were studied. Selected molecules associated with the STAT3 oncogenic pathway, including pSTAT3, Bcl-xL, survivin, cyclin D1 and Ki-67, were further analyzed. A total of 135 OL lesions, including 97 PLs and 38 recurrences, were included. Out of 97 PLs, 31 recurred at least once and none of them underwent MT, during a mean follow-up time of 48.3 months. There was no statistically significant difference among the various parameters in recurrent vs. non-recurrent PLs, although recurrence was most frequent in non-homogeneous lesions (*p* = 0.087) and dysplastic lesions recurred at a higher percentage compared to hyperplastic lesions (34.5% vs. 15.4%). Lower levels of Bcl-xL and survivin were identified as significant risk factors for OL recurrence. Recurrences, although smaller and more frequently homogeneous and non-dysplastic compared to their corresponding PLs, exhibited increased immunohistochemical expression of oncogenic molecules, especially pSTAT3 and Bcl-xL. Our results suggest that parameters associated with recurrence may differ from those that affect the risk of progression to malignancy and support OL management protocols favoring excision and close monitoring of all lesions.

## 1. Introduction

Oral cancer is one of the most frequent types of cancer worldwide, accounting for approximately 380,000 new cases annually, with oral squamous cell carcinoma (OSCC) being the most common type [1]. Despite the achieved progress in treatment modalities, OSCC is associated with significant morbidity and mortality; despite geographic variations, the 5-year overall survival rate in general remains dismal, estimated at around 50% in most studies [2]. Therefore, there is an urgent need for improvement though primary prevention (aiming at avoidance of exposure to known risk factors, such as smoking and alcohol), as well as secondary prevention with emphasis on early detection and proper management of OSCC precursor lesions [3].

It is generally accepted that OSCC may arise either de novo or, most often, in the context of a preceding oral potentially malignant disorder (OPMD), most commonly oral leukoplakia (OL). According to the recent recommendation by the WHO Collaborating Centre for Oral Cancer, OPMDs can be defined as “any oral mucosal abnormality that is associated with a statistically increased risk of developing oral cancer.” [4]. OPMDs encompass a spectrum of oral mucosal diseases (e.g., OL, erythroplakia, oral lichen planus, oral submucous fibrosis etc.) with a global prevalence of 4.47%, albeit with variable rates among different types of lesions and populations, as has been estimated in a recent systematic review [5]. Among OPMDs, OL is the most common with a worldwide prevalence of 4.11% [5]. Several definitions of OL have been used throughout the years; according to the WHO, “Leukoplakia is a clinical term used to describe white plaques of questionable risk, once other specific conditions and other OPMDs have been ruled out” [6]. OL may undergo malignant transformation (MT) at a rate ranging widely in various studies between 0.13–34%, with an annual progression rate of 1–3% [7,8,9]. Moreover, its co-existence in about 50% of OSCC cases at the time of diagnosis supports the necessity for early diagnosis and prevention of MT of OL, especially considering the dismal prognosis of OSCC [10,11]. Besides MT, OL recurrences, sometimes multiple, are generally regarded as common with a 5-year incidence of almost 50% [12]. Although it is intuitive that OL recurrences pose a significant problem, indicating persistence and suggesting tendency for progression and also raising significant management concerns, they have not been studied as thoroughly so far.

Various sociodemographic factors (older age, female gender, non-smoking), clinical characteristics (increased size, non-homogeneous appearance, high risk sites such as lateral and ventral tongue and floor of mouth), and histopathologic features (higher grades of dysplasia) have been shown to correlate with risk of progression [13]. Additionally, special attention has been given to the potential usefulness of molecular markers in predicting oral cancer development in OPMDs [14,15]. However, to this date, there are still important limitations, since no single marker that could estimate the precise malignant potential of OL has been corroborated yet, urging for further studies that may shed light on the molecular nature of OL [14,16,17]. This is supported by a recent systematic review [18] of biomarkers in OL failing to generate conclusive results due to the heterogeneity of studies. Most of the aforementioned studies have correlated these features with MT, while very few studies, as noted above, have specifically focused on OL recurrence, attempting to identify parameters that could determine the risk of relapse, following removal of the initial lesions [12,19].

In the present study, we aimed to further investigate the phenomenon of recurrence in OL. Specifically, in a well-characterized cohort of 33 patients with one or more OL lesions, which were all subjected to excisional biopsy or incisional biopsy and laser ablation and then submitted to adequate follow-up, we first recorded all recurrences, new primary OL lesions (PLs) or OSCCs developing during follow-up. We also evaluated the demographic, clinical, and histopathologic characteristics of PLs and correlated them with their risk of recurrence after treatment. Further, we assessed the immunohistochemical (IHC) expression of selected cell cycle and apoptosis-related molecules (associated with the Stat3 oncogenic pathway, including pSTAT3, Bcl-xL, survivin, cyclin D1 and Ki-67) [20] and correlated them with the presence and degree of epithelial dysplasia, as well as with the risk of OL recurrence. Finally, we compared the aforementioned clinical, microscopic, and IHC features between PLs and their respective recurrences observed during follow-up.

## 2. Materials and Methods

### 2.1. Patients’ Records

The clinical charts of 33 patients, managed at the Oral Medicine Clinic of the Department of Oral Medicine & Pathology and Hospital Dentistry, School of Dentistry, National and Kapodistrian University of Athens (NKUA), Greece, between January 2010 and March 2020, were reviewed. All patients had an initial clinical diagnosis of OL, which was submitted to biopsy and histopathologically confirmed as either epithelial hyperplasia or epithelial dysplasia of mild, moderate, or severe degree [6,21].

Inclusion criteria encompassed availability of demographic data, including age, gender, smoking habits, alcohol consumption, and complete medical history; detailed clinical information, including number, site, size and clinical appearance (homogeneous vs. heterogeneous) of lesions; biopsy (incisional or excisional) and histopathologic diagnosis with tissue availability; and close follow-up for at least 1 year.

Exclusion criteria were a previous diagnosis of head and neck SCC in the last 18 months before first visit and a history of previous chemo- or radiotherapy. White non-removable lesions consistent with frictional keratosis or other diagnoses (different than OL) were also excluded from the study.

According to our institutional regulations, this study was exempted from institutional review board approval.

### 2.2. Demographic Data

Data collected included epidemiologic characteristics, such as age and gender. Smoking and drinking habits were also recorded and patients were classified as either non-smokers (those who had never smoked) and ex-smokers (when a person had quit smoking at least 1 year prior to diagnosis), or as current smokers (those that are currently smoking or had stopped smoking <1 year prior to diagnosis) [22]. Based on alcohol consumption, patients were classified as drinkers vs. non-drinkers, the latter category including never drinkers and social drinkers [23].

### 2.3. Clinical Features

The available clinical parameters including number (uni- or multifocality), site and size of the lesions were recorded. Based on their clinical appearance, OL lesions were classified as homogeneous or non-homogeneous; the latter category included verrucous, speckled (erythro-leukoplakia) and nodular lesions [6,24]. Clinical photographs were available in all cases.

### 2.4. Biopsy and Histopathologic Features

All lesions included in our study had been submitted either to an excisional biopsy, when lesions were sized approximately ≤1 cm, or an incisional biopsy (size > 1 cm) with scalpel (performed under local anesthesia by an experienced clinician following similar technique). For excisional biopsies, a 3 mm margin of normal tissue was included.

Two oral and maxillofacial pathologists (N.N, A.L) re-evaluated the hematoxylin and eosin-stained (H&E) sections of all lesions for confirmation of the diagnosis. A microscopic diagnosis of epithelial hyperplasia or dysplasia of mild, moderate or severe degree was rendered according to the WHO classification [6,21]. For excisional biopsies, the status of the surgical margins, i.e., positive vs. negative, was also recorded.

### 2.5. Management

All cases removed by excisional biopsy were placed in follow-up. All cases first submitted to incisional biopsy were subsequently subjected to CO_2_ laser ablation with approximately 3 mm margin (beyond the clinically visible border of the residual OL in all directions). Small lesions were treated in one laser session; larger lesions needed two or more treatments.

The CO_2_ laser system was the Smart US20 D laser class 4 (DEKA M.E.L.A, Calenzano, Italy). The standard laser protocol, used in the Department of Oral & Maxillofacial Surgery, School of Dentistry, National and Kapodistrian University of Athens, Greece, was applied. Local anesthesia was administered peripherally to the borders of the lesion. The contour of the lesion was marked out in a pulsed mode with the laser, including an approximately 3 mm area of normal appearing mucosa peripherally to the lesion. The light ray of the laser during the sublimation was at a distance of about 1 mm, the wavelength ranged between 250–500 µm at 2–5 watts in continuous wave, and the pulse frequency was of about 100 Hz. All wounds caused by the laser were left open for secondary intention healing. All patients attended regular follow-up after treatment to evaluate the healing process.

### 2.6. Follow-Up

Only patients seen at regular intervals for at least 1 year were included in the study. Patients were re-examined at least every 2 months until the end of the first year and subsequently on a 3-month basis. Clinical photographs of the PLs, taken with a graduated ruler in the vicinity, were available and compared in order to facilitate recognition of a clinical recurrence.

Local recurrence was defined as an OL lesion arising in the immediate vicinity of the treated area. In these cases, clinical parameters of the new lesion (size, site, homogeneity or not) were also carefully recorded and a new biopsy (incisional or excisional, according to size, similar to PLs) was performed under local anesthesia. Following histopathologic confirmation of the diagnosis (epithelial hyperplasia or dysplasia of various degrees), laser ablation (of the recurrent lesions first submitted to incisional biopsy) was performed, as described above.

In addition to local recurrences, all new PLs (i.e., at different sites compared to the original PLs) arising during the follow-up period were also detected and recorded according to their clinical features, submitted to biopsy (incisional or excisional, according to size) and histo-pathologically evaluated and managed, following the same protocol as for the original PLs noticed at first visit.

### 2.7. Immunohistochemistry Experiments

From each tissue block, four-µm-thick serial formalin-fixed paraffin-embedded tissue sections of biopsy specimens were deparaffinized and placed on charged slides. The slides were incubated in xylene and then immersed in ethanol 100% and 95% and heated for antigen retrieval in 0.01 M citrate buffer (C2488, Sigma-Aldrich, St. Louis, MO, USA) for 25 min in a pressure cooker inside a microwave oven. Endogenous peroxidase activity and non-specific protein reaction were then blocked. After dehydration in hydrogen peroxide, the sections were incubated with primary antibodies at room temperature for 1 h. The applied antibodies were all rabbit monoclonal against phospho-STAT3 (pSTAT3) (Tyr705) (CST, D3A7, #9145) (1:200), survivin (CST, 71G4B7, #2808) (1:300), cyclin D1 (CST, E3P5S, #55506) (1:300), Ki-67 (CST, D2H10, #9027) (1:250), and Bcl-xL (CST, 54H6, #2764) (1:200) (Cell Signaling Technology, Leiden, The Netherlands).

The standard streptavidin-biotin-peroxidase complex method was employed to bind to the primary antibody along with mouse anti-rabbit IgG as secondary antibody (1:2000) (CST#7074, Cell Signaling Technology, Leiden, The Netherlands). Reaction products were visualized by counterstaining with the 3,3V-diaminobenzidine reagent set (Kirkegaard and Perry Laboratories, Gaithersburg, MD, USA). Sections were counterstained with hematoxylin. As a negative control, sections were treated with phosphate-buffered saline (PBS) with omission of the primary antibody. Immunostains were reviewed by three evaluators (N.N., E.P., D.V.).

To validate the staining in all samples, positive controls of tissue sections known to express the five studied proteins were used (including breast cancer for phospho-STAT3, prostate cancer for survivin and Bcl-xL, papillary carcinoma of thyroid gland for cyclin D1, and small cell carcinoma of the bladder for Ki-67).

Positive and negative controls were included in every IHC run to ensure that technical variation did not affect the results.

Some samples were not evaluated, because of progressive depletion of the representative areas of the sample material.

### 2.8. Evaluation of Immunohistochemical Staining

The IHC staining was evaluated by three independent evaluators (N.N., E.P., D.V.). Sections were scored as positive if the epithelial cells showed immunoreactivity in the nucleus.

The tissue sections were scored based on the percentage of positive cells in a semiquantitative manner: (0) <1%; (1) 1–25%; (2) 26–50%; (3) >50%. Sections were also scored on the basis of staining intensity as (0) no staining, (1) mild, (2) moderate, or (3) strong, compared with the positive control tissues, the intensity of which was classified as moderate. Lower intensity (light brown) compared to the brown staining of the positive control was classified as weak, while higher intensity (dark brown) compared to the positive control was classified as strong. Finally, a total score (0, 2–6) was obtained by adding the scores of percentage of cells positivity (0–3) and intensity (0–3). At least five random high-power magnification fields of view (of at least 100 total cells each) of selected areas (representative of the final diagnosis) of each specimen were analyzed independently and the average scores were calculated [25]. In the limited number of cases that an initial disagreement among the independent evaluators was noticed, a consensus agreement was achieved by the use of a multi-observer microscope.

All images were scanned and photographed using the WSI software program (Microvisioneer, Germany) with an Olympus CX 23 microscope.

### 2.9. Data Comparison and Statistical Analysis

Comparative evaluation was performed as follows:

- Recurrent vs. non-recurrent PLs were compared according to demographic data and patients’ habits (age, gender, smoking, and alcohol use), clinical features (site, size, homogeneity or not) and histopathologic features (epithelial hyperplasia vs. dysplasia of various degrees). For small lesions removed by surgical excisional biopsy, comparison was also made according to the histopathologic status of the surgical margins.
- The IHC scores of the five molecules investigated were also compared between recurrent vs. non-recurrent primary OL lesions, as well as between lesions with different histopathologic diagnoses (epithelial hyperplasia vs. dysplasia of various degree).
- For lesions developing recurrence, all the above parameters were also compared between PLs and their corresponding recurrent lesions.

The baseline characteristics were summarized as absolute (*n*) and relative (%) frequencies in tables and relevant data were compared in terms of two tailed Fisher’s exact test. Statistical comparisons of molecular marker IHC scores were performed with Mann-Whitney U test, Kruskal-Wallis H test and Wilcoxon Signed Rank test. Disease free survival (DFS) curves were calculated according to the method of Kaplan and Meier. The significance of all parameters under study for DFS was analyzed by the Cox proportional hazards model. Statistical analyses were performed using the SPSS software application (version 21.0: SPSS, Chicago, IL, USA.) with *p* < 0.05 as the threshold of significance.

## 3. Results

### 3.1. Patients’ Demographic Data and Follow-Up

A total number of 33 patients with OL were included in the present study; 21 were females and 12 males (female to male ratio: 1.75:1). Patients’ age at inclusion ranged from 35.8 to 88.4 years with a mean age of 58.3; most individuals (11/33, 33.3%) belonged to the 55–65 age group. Concerning smoking, 19/33 (57.6%) were smokers, 9/33 (27.3%) were ex-smokers, while 5/33 (15.2%) were non-smokers. Regarding alcohol use, 7/33 (21.2%) were drinkers, while 26/33 were non-drinkers and social drinkers (78.8%).

Follow-up for all patients extended for at least 12 months ranging from 12.0 to 108.7 months (median: 48.3 months).

### 3.2. Distribution of Lesions

#### 3.2.1. OLs

**Figure 1:**
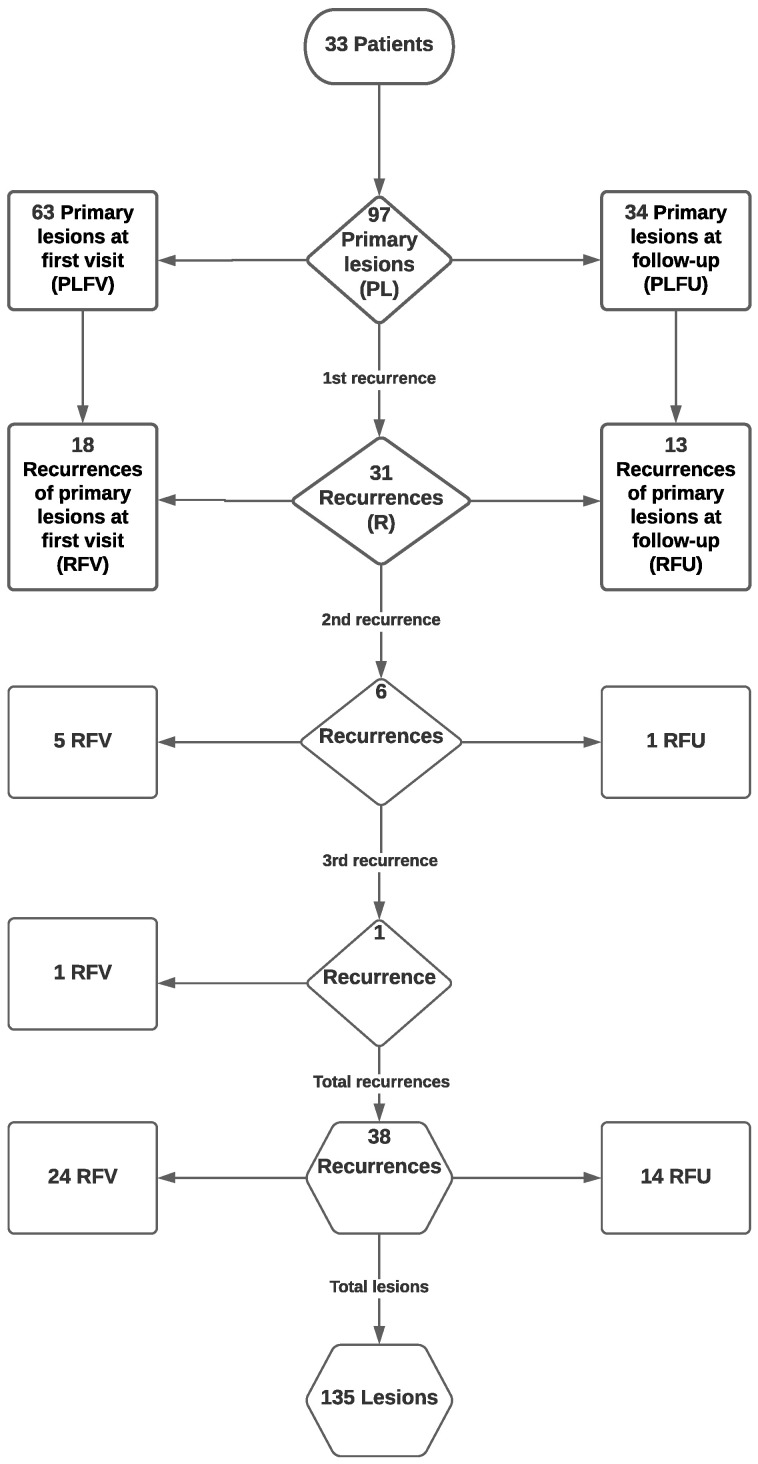
Flowchart of all 135 oral leukoplakia (OL) lesions, developing primary lesions (PLs) and recurrences (Rs), in all 33 patients.

A total of 135 OPMD lesions, all classified as OLs, were recorded in all 33 patients. Specifically, the OL lesions were categorized as follows (Figure 1):

1. Primary lesions noticed at first visit (PLFV): a total of 63 lesions
2. Primary lesions noticed at follow-up (PLFU): a total of 34 lesions
3. All primary lesions (PL): a total of 97 lesions
4. Recurrences of primary lesions noticed at first visit (RFV): a total of 24 lesions
5. Recurrences of primary lesions noticed at follow-up (RFU): a total of 14 lesions
6. All recurrences: a total of 38 lesions

Therefore, out of 135 OL lesions, 97 (71.9%) were PLs and 38 (28.1%) were recurrences. Concerning PLs, 63 (64.9%) were present at patient’s inclusion and 34 (35.1%) were first noticed during follow-up.

#### 3.2.2. OSCC

Only one OSCC lesion was recorded in all 33 patients. Specifically, this lesion was not present at first visit, but developed as a new lesion in the floor of the mouth of a patient at 33 months of follow-up; the patient was male, smoker, drinker and, at first visit, he was 67 years old. He presented with a homogeneous OL in the buccal mucosa, which was diagnosed as mild dysplasia and removed by laser ablation; neither recurrence of the primary OL nor development of new primary OL lesions were noticed. On the other hand, none of the primary or recurrent OL lesions underwent MT.

### 3.3. Characteristics of PLs

The characteristics of all 97 PLs (including 63 noticed at first visit PLFV and 34 discovered during follow-up PLFU) are summarized in Table 1. Briefly:

- The most commonly affected site was the buccal mucosa [32/97 (33%) of PLs, including 24/63 (38.1%) PLFVs and 8/34 (23.5%) PLFUs], followed by lower gingiva/alveolar mucosa [PLs: 22/97 (22.7%), PLFVs: 14/63 (22.2%), PLFU: 8/34 (23.5%)].
- Regarding size of lesions, the mean largest dimension was 1.4 (±0.7) for PLs, 1.5 (±0.7) for PLFVs and 1.2 (±0.5) for PLFUs.
- Regarding OL homogeneity, among 97 PLs, 71 (73.2%) were homogeneous and 26 (26.8%) were non-homogeneous. In PLFVs, 49/63 (77.8%) were homogeneous and 14/63 (22.2%) were non-homogeneous, while in PLFUs, 22/34 (64.7%) were homogeneous and 12/34 (35.3%) were non-homogeneous.
- Regarding treatment method, 52 (53.6%) PLs were removed by laser ablation (following incisional biopsy) and 45 (46.4%) were surgically removed (by excisional biopsy).
- Regarding histopathologic subtype, most PLs were classified as mild dysplasias (61/97, 62.9%), followed by moderate dysplasias (21/97, 21.6%), hyperplasias (13/97, 13.4%) and severe dysplasias (2/97, 2.1%). Similarly, in PLFVs and PLFUs, the majority of cases were histo-pathologically diagnosed as mild dysplasias (38/63, 60.3% and 23/34, 67.6%, respectively).

### 3.4. Number, Types and Characteristics of Recurrences

**Figure 2:**
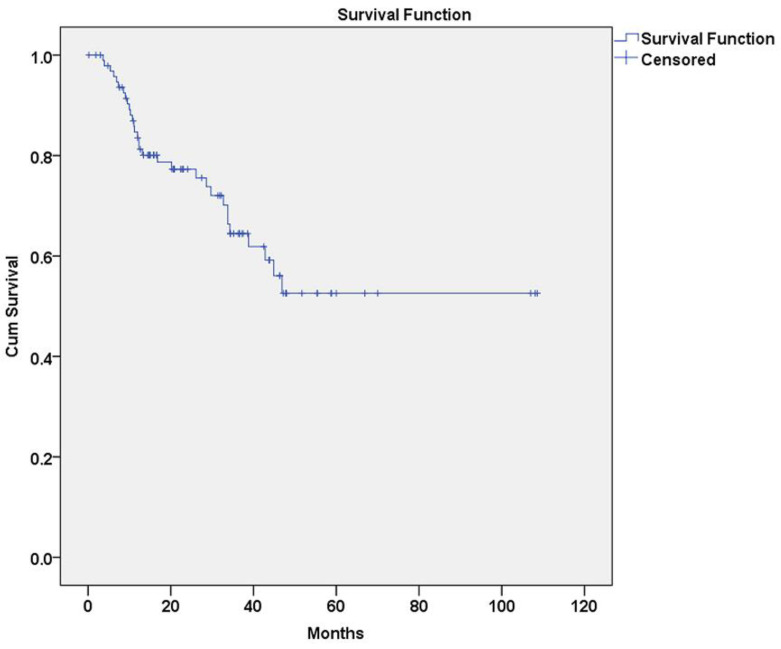
Kaplan–Meier analysis of first recurrence in oral leukoplakia (OL) lesions (*n* = 31).

A total of 38 recurrent lesions were recorded and characterized as first (*n* = 31, 81.6%), second (*n* = 6, 15.8%) and third (*n* = 1, 2.6%) (Figure 1). First recurrences appeared between 3.6 and 46.9 months after initial treatment, second recurrences appeared between 3.6 and 49.3 months after previous treatment and the sole third recurrence appeared in 26.2 months after previous treatment. Excluding the sole OSCC lesion, mean disease-free survival time (DFS) between removal of PL and first recurrence was 68.9 months (95% CI: 57.5–79.8 months) (Figure 2).

The characteristics of all 38 recurrent lesions are summarized in Table 1. Briefly:

- Regarding site, most recurrent lesions were located in the buccal mucosa (12/38, 31.6%), followed by lower gingiva/alveolar mucosa (9/38, 23.7%).
- Regarding size of lesions, the mean largest dimension was 0.9 (±0.4).
- Regarding homogeneity, 32 (84.2%) were homogeneous and 6 (15.8%) were non-homogeneous.
- Regarding treatment, 13 (34.2%) recurrent lesions were removed by laser ablation (following incisional biopsy) and 25 (65.8%) were surgically removed (by excisional biopsy).
- Regarding histopathologic subtype, most recurrent lesions were classified as mild dysplasias (19/38, 50.0%), followed by hyperplasias (11/38, 28.9%), moderate dysplasias (7/38, 18.4%), and a single case of severe dysplasia (1/38, 2.6%).

### 3.5. Comparison of Demographic, Clinical and Histopathologic Data between Recurrent and Non-Recurrent PLs

Frequency distribution and comparison of recorded variables between recurrent and non-recurrent PLs are shown in Table 2. Out of 97 PLs, 66 (68.0%) did not recur and 31 (32.0%) recurred (once or more) with a histopathologically confirmed diagnosis of OL; as noted before, none of the PLs underwent MT.

There was no statistically significant difference among the various parameters in the recurrent vs. non-recurrent group. Specifically, no significant gender or age differences were noted, although females accounted for a relatively higher percentage of PLs that recurred (61.3%) compared to non-recurrent (54.5%). Similarly, smoking or alcohol consumption habits did not differ significantly among recurrent and non-recurrent PLs.

Regarding location, the majority of 31 PLs that recurred were located in the buccal mucosa: specifically, recurrences occurred in 9/31 (29.0%) PL lesions located in the buccal mucosa (two of which recurred twice or thrice, respectively). In addition, recurrences were noted in 7/22 (31.8%) PLs located in lower gingiva/alveolar mucosa, 5/13 (38.5%) PLs located in upper gingiva/alveolar mucosa, 4/6 (66.7%) PLs located in hard palate, 4/12 (33.3%) of PLs located in ventral tongue/floor of the mouth and 2/13 (15.4%) of PLs located in lateral/dorsal tongue. However, no significant differences in site were recorded among recurrent vs. non-recurrent PLs.

As far as size was concerned, the percentage of larger lesions (≥2.0 cm) was higher among recurrent compared to non-recurrent PLs (35.5% vs. 24.2%, respectively); however, this difference was not statistically significant. Interestingly, non-homogeneous lesions were more common among recurrent vs. non-recurrent PLs (38.7% vs. 21.2%, respectively); this difference approached, but did not reach, statistical significance (*p* = 0.087). Treatment method (surgical excision vs. laser ablation) was not different between the two groups.

With regards to histopathologic diagnosis, no statistically significant differences were noticed between the two groups, although hyperplasias were less frequent in recurrent vs. non-recurrent PLs (6.5% vs. 16.7%, respectively). For lesions managed by surgical excision, there were no significant differences in margin status (positive vs. negative) between the two groups.

### 3.6. IHC Analysis of Molecular Markers and Correlation with Degree of Dysplasia

**Figure 3:**
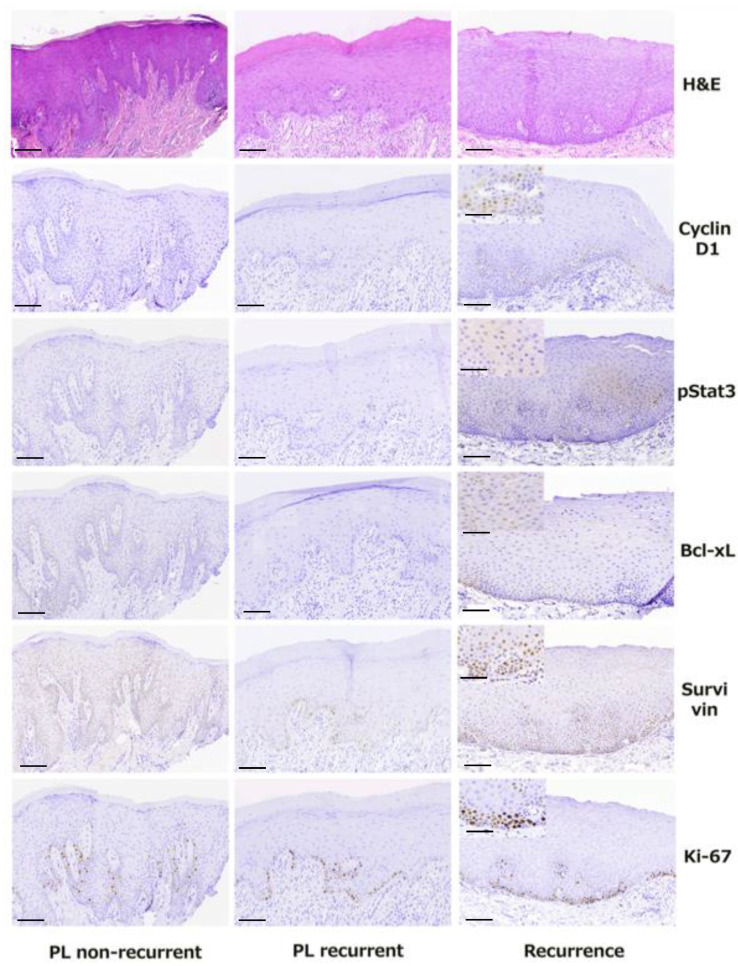
Representative photomicrographs of hematoxylin and eosin (H&E) and immunohistochemical stains of all studied molecules (cyclin D1, pSTAT3, Bcl-xL, survivin and Ki-67) in a non-recurrent primary oral leukoplakia lesion (PL)–1st column, in a recurrent PL–2nd column and in its corresponding recurrence–3rd column (magnification 100×, scale bar 100 μm; for inserts magnification 200×, scale bar 50 μm).

For IHC analysis, selected molecules modulated by the oncogenic STAT3 signaling pathway and associated with cell proliferation and/or apoptosis were studied, on the basis of previously published evidence suggesting their potential prognostic and predictive values in the context of OPMDs [14,20]. The results are summarized in Table 3 and illustrated in Figure 3.

#### 3.6.1. Cyclin D1

IHC for cyclin D1 demonstrated positivity with nuclear localization in the vast majority of studied cases (97.1%), showing variable intensity and percentage of positive cells (Figure 3).

Regarding PLs, 96.5% of lesions were positive with the mean percentage, intensity and total scores being 0.97, 1.44 and 2.41, respectively (Table 3); for PLFVs vs. PLFUs, the corresponding mean scores were 0.95, 1.43 and 2.38 vs. 1.00, 1.48 and 2.48, respectively.

**Figure 4:**
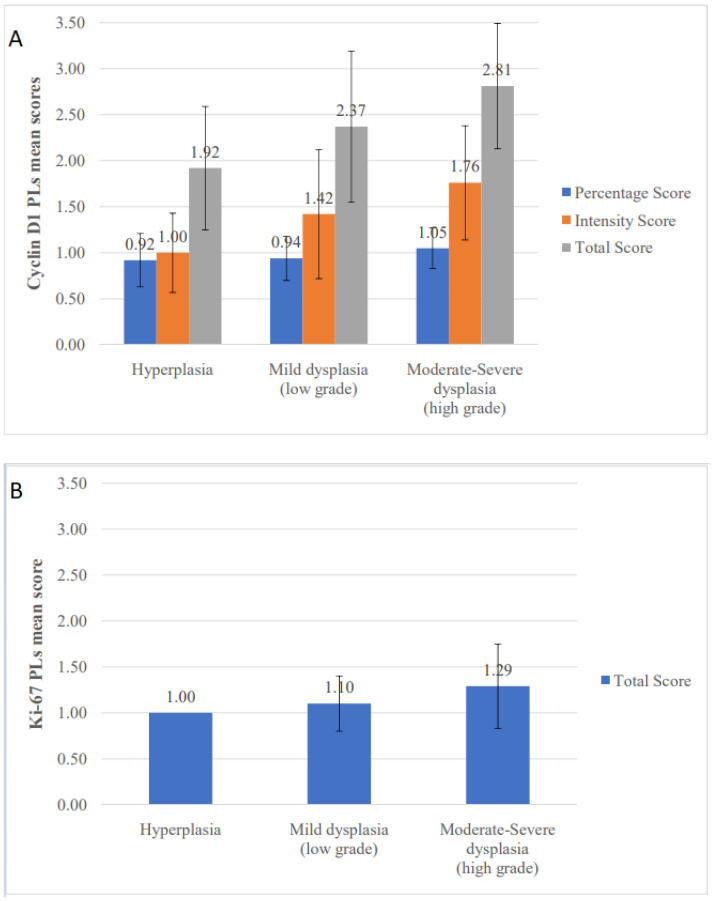

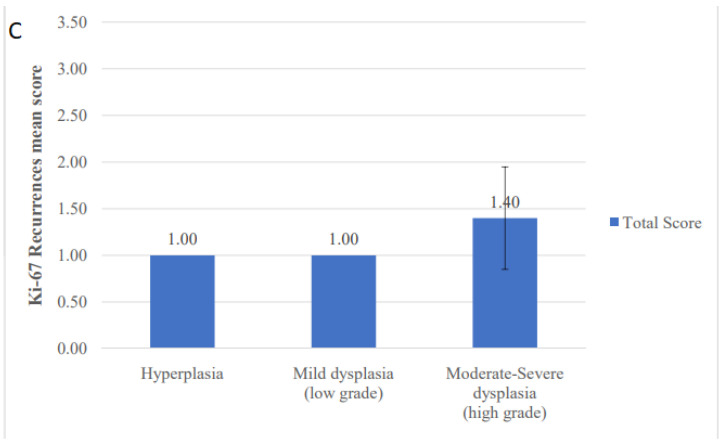
Comparison of mean immunohistochemical scores according to the presence and degree of dysplasia. (**A**) Cyclin D1 percentage, intensity and total scores in primary oral leukoplakia lesions (PLs), (**B**) Ki-67 total score in PLs and (**C**) Ki-67 total score in recurrences.

Cyclin D1 IHC scores in PLs were further analyzed according to the presence and degree of dysplasia. It was noticed that high grade dysplasias (moderate and severe) received higher mean total scores compared to hyperplasias and mild dysplasias (Figure 4A). Statistically significant differences were found for intensity and total scores (*p* = 0.004).

Regarding recurrences, all cases were positive with mean percentage, intensity and total scores of 1.00, 1.84 and 2.84, respectively (Table 3). No significant differences were found among different grades of dysplasia for recurrent lesions.

#### 3.6.2. pSTAT3

pSTAT3 IHC positivity in the nucleus was detected in the majority of studied cases (64.4%), manifesting variations in intensity and percentage of positive cells (Figure 3).

Regarding PLs, 61.2% of cases were positive with mean percentage, intensity and total scores of 0.84, 0.88 and 1.72, respectively (Table 3); for PLFVs vs. PLFUs, the corresponding mean scores were 0.87, 0.90 and 1.77 vs. 0.80, 0.88 and 1.68, respectively. There were no statistically significant differences related to presence and degree of dysplasia among PLs.

Regarding recurrences, a higher percentage (78.9%) of positive cases was detected compared to PLs; the mean percentage, intensity and total scores were 1.26, 1.63 and 2.89, respectively (Table 3). Classifying recurrences on the basis of the presence and degree of dysplasia did not reveal any significant differences.

#### 3.6.3. BCL-xL

BCL-xL exhibited IHC positivity with nuclear localization in the majority of cases (84.6%), showing variable intensity and percentage of positive cells (Figure 3).

Regarding PLs, 82.4% of cases were positive with mean percentage, intensity and total scores of 1.47, 1.29 and 2.76, respectively (Table 3); the corresponding mean scores were 1.57, 1.38 and 2.95 for PLFVs vs. 1.16, 1.00 and 2.16 for PLFUs, respectively. Separating PLs based on the presence and degree of dysplasia did not render any statistically significant differences.

Regarding recurrences, the percentage of positive cases was 94.7% (higher compared to PL cases); the mean percentage, intensity and total scores were 1.79, 1.79 and 3.58, respectively (Table 3), without any significant differences associated with the presence or degree of dysplasia.

#### 3.6.4. Survivin

Immunostaining for survivin showed nuclear positivity of variable percentage of positive cells and intensity and in the vast majority of studied cases (96.2%) (Figure 3).

In PLs, almost all lesions were positive (98.8%), the mean percentage, intensity and total scores being 2.17, 2.26 and 4.43, respectively (Table 3); the corresponding mean scores in PLFVs vs. PLFUs were 2.18, 2.30 and 4.48 vs. 2.12, 2.12 and 4.24, respectively. No statistically significant differences were found among PLs, according to the presence and degree of dysplasia.

For recurrences, positive cases accounted for 84.2%; the mean percentage, intensity and total survivin scores were 1.89, 1.84 and 3.74, respectively (Table 3). Again, no significant differences were found, when recurrences were classified according to the presence and degree of dysplasia.

#### 3.6.5. Ki-67

IHC for the cell proliferation index Ki-67 showed variable positivity (i.e., percentage of positive nuclei) in all cases (Figure 3).

Regarding PLs, PLFVs and PLFUs, the mean percentage score was 1.14, 1.15 and 1.08, respectively.

When PLs were further analyzed according to the presence and degree of dysplasia, high grade dysplasias (moderate and severe) received higher mean scores compared to hyperplasias and mild dysplasias (Figure 4B). A statistically significant difference was found (*p* = 0.034).

Regarding recurrences, the mean percentage score was 1.11. Similar to PLs, higher grades of dysplasia received higher scores compared to hyperplasias and mild dysplasias (Figure 4C). The difference almost reached statistical significance (*p* = 0.052).

### 3.7. Comparison of IHC Expression Levels between Recurrent and Non-Recurrent Primary Lesions

**Figure 5:**
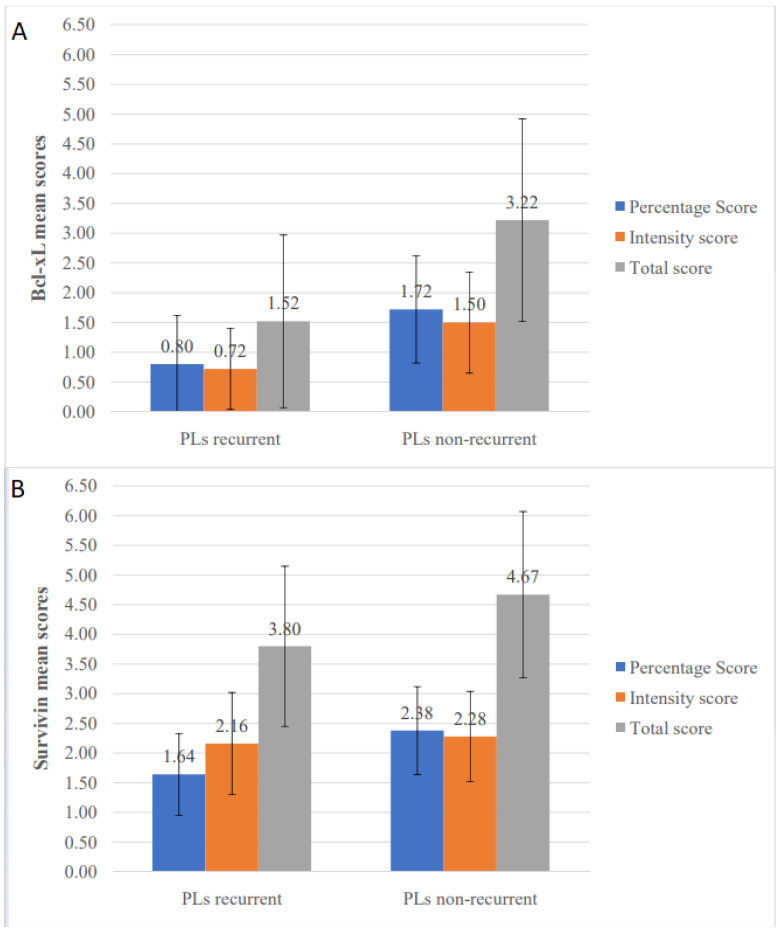
Comparison of immunohistochemical mean scores between recurrent and non-recurrent primary oral leukoplakia lesions (PLs). (**A**) Bcl-xL percentage, intensity and total scores and (**B**) survivin percentage, intensity and total scores.

In order to assess the potential usefulness of the studied IHC markers as predictors of recurrence of PLs, the percentage, intensity and total scores of each molecule were compared between recurrent and non-recurrent PLs (Table 4 and Figure 5). Statistically significant differences were noticed only for Bcl-xL and survivin. Specifically, Bcl-xL received lower values in the recurrent group for percentage (*p* < 0.001), intensity (*p* < 0.001), and total scores (*p* < 0.001). Similarly, survivin percentage (*p* < 0.001) and total scores (*p* = 0.006) were lower in PLs that recurred compared to non-recurrent ones.

### 3.8. Univariate and Multivariate Analysis of Predictors of Recurrence in OLs

The results from the univariate Cox regression analysis revealed that a lower survivin percentage score, as well as lower Bcl-xL intensity, percentage and total scores were significant risk factors for recurrence of PLs (Table 5); no other parameters (including demographic data, patient habits, clinical or histopathologic parameters) reached statistical significance. Multivariate stepwise backward Wald Cox regression analysis showed that lower Bcl-xL percentage score remained as a significant risk factor for recurrence in OPMDs (HR: 0.442, 95% CI: 0.271–0.720, *p* = 0.001). The Cox proportional hazards assumption was fulfilled for all the analyzed variables.

### 3.9. Comparison of Clinical, Histopathologic and IHC Features between PLs That Recurred and Their Corresponding Recurrences

In order to evaluate the evolution of various lesional characteristics during recurrence, we compared clinical, histopathologic and IHC parameters between PLs that recurred and their corresponding recurrences (Table 6 and Table 7).

Clinical parameters compared between PLs and their first recurrences included size and homogeneity (Table 6). Regarding size, statistically significant difference (*p* < 0.001) was noticed, corresponding to the fact that all recurrent lesions were small in size (<2.0 cm), compared to 64.5% of PLs with similar size. Regarding homogeneity, 19/31 (61.3%) of PLs that recurred were homogeneous, in comparison with 27/31 (87.1%) homogeneous recurrences; this difference was also statistically significant (*p* = 0.040).

With regards to histopathologic diagnosis, statistically significant differences (*p* = 0.028) were noticed between PLs and their recurrences (Table 6). More specifically, most recurrent PLs were classified as mild (24/31, 77.4%) or moderate dysplasias (5/31, 16.1%) with only 2/31 (6.5%) being diagnosed as hyperplasias; in contrast, in their corresponding first recurrences, hyperplasias accounted for a higher percentage (10/31, 32.3%), while mild dysplasias were relatively fewer (15/31, 48.4%) compared to PLs; interestingly, moderate dysplasia cases were the same (5/31, 16.1%), while one single case (3.2%) of severe dysplasia was recorded among recurrences (its corresponding lesion being diagnosed as mild dysplasia).

**Figure 6:**
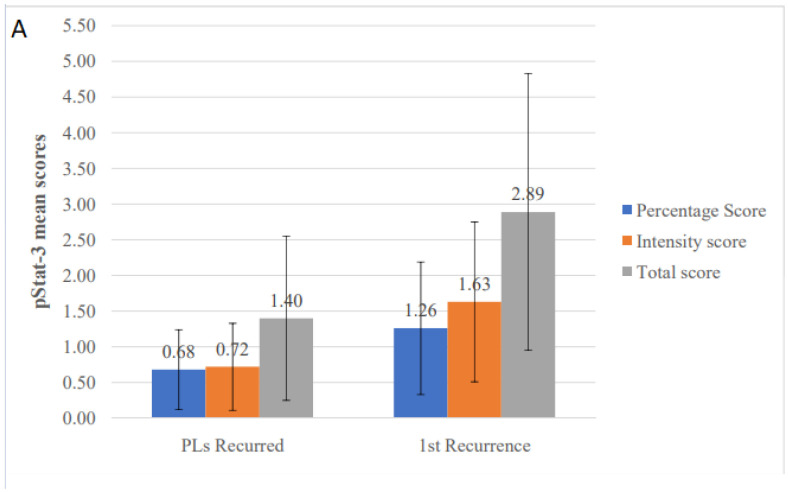

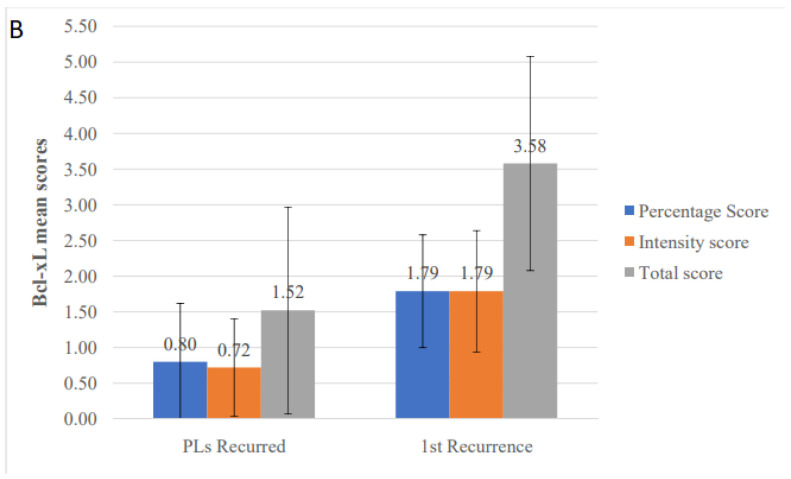
Comparison of immunohistochemical mean scores of (**A**) pSTAT3 and (**B**) Bcl-xL, between primary oral leukoplakia lesions (PLs) that recurred and their corresponding first recurrences.

When IHC expression levels of various studied molecules were compared between the two groups, higher mean total values were seen in the recurrences compared to their corresponding PLs for cyclin D1 (2.84 vs. 2.36), pSTAT3 (2.89 vs. 1.40) and Bcl-xL (3.58 vs. 1.52), but not for survivin (3.74 vs. 3.80) and Ki-67 (1.11 vs. 1.20). For pSTAT3, both total (*p* = 0.018) and intensity (*p* = 0.009) scores were statistically significant higher in recurrences with percentage score showing a tendency towards significance (*p* = 0.054) (Table 7 and Figure 6A). Similarly, significantly higher values for Bcl-xL were noticed in recurrences compared to PLs for percentage (*p* = 0.007), intensity (*p* = 0.006) and total (*p* = 0.005) scores (Table 7 and Figure 6B). No significant differences among PLs and their recurrences were found for the other molecules, although cyclin D1 total score and survivin intensity score almost reached statistical significance (*p* = 0.070 and *p* = 0.059, respectively) (Table 7).

## 4. Discussion

In this descriptive analysis of well-documented OL cases, we investigated key demographic, clinical, microscopic, and IHC parameters that could affect its biologic behavior. Malignant transformation (MT) is justifiably considered as the most important end point of the behavior of this entity and, as a result, several comparative studies have assessed various parameters affecting the risk of transition from OL to OSCC [4,26]. On the other hand, few studies have focused on OL recurrence, although it is evident that it represents an important factor affecting the outcome of these lesions [12,19], especially taking into consideration that development of OSCC (including progression of pre-existing OPMDs) does not usually occur abruptly, but involves a multistep process driven by the gradual accumulation of molecular defects [14]. Hence, it is fundamental also to characterize OL recurrences, including their rate, pattern and potential predictors, since they could be intimately associated with a tendency towards MT [12,27]. The clinical significance of assessing the risk of recurrence and progression is its potential utility for developing individualized protocols of management and follow-up, based on the various clinicopathologic and IHC findings of each specific case.

In the present study, we closely followed-up 33 patients with OL, following lesions removal either by incisional biopsy and laser ablation or scalpel surgical excision. We defined recurrence as the development of a new lesion in the same anatomic location of a previously treated OL We separately assessed the lesions that developed subsequently during follow-up in other oral anatomic sites, also recording their potential recurrences. An alternative consideration could be to regard these new distant lesions as recurrences, since OPMDs could be construed as generalized disorders rather than localized lesions [6]; however, we preferred to consider all lesions separately, acknowledging the possibility that multifocal lesions, developing synchronously or metachronously, could be also attributed to independent genetic events and not necessarily to a widespread field cancerization effect.

Noticeably, cancer development occurred in only one case and no patient developed OSCC in the same anatomic region as the initial OL; hence, statistical correlations of the various parameters with subsequent MT could not be carried out. This decreased number of MT in our study, despite adequate follow-up, could be attributed to the strict protocol used, involving very frequent recall appointments, meticulous oral examination by specialized clinicians, and prompt diagnostic and management intervention (including new biopsies, either excisional or incisional followed by laser ablation) for all new lesions detected. On the contrary, recurrence occurred in 18/63 PLs noticed at first visit; of these, four lesions recurred twice and one lesion recurred three times. In addition, during follow-up, 34 new primary OLs (defined as new lesions developing at different sites compared to the original primary OLs) were detected, out of which 13 recurred (once or, in one case, twice). Therefore, in the whole sample of 97 PLs, all of which were removed by surgical excision or laser ablation with adequate margin, a significant percentage (32% or approximately one in every three lesions) recurred once or more.

Holmstup et al. [27] reported a 12.8% recurrence rate of OPMDs (12/94) following scalpel surgical removal, including 2/39 (5.1%) homogeneous OL, 8/46 (17.4%) non-homogeneous OL and 2/9 (22.2%) erythroplakias. Similar to these findings, we noted more frequent recurrences in non-homogeneous OLs (12/26, 46.2%) vs. homogeneous OLs (19/71, 26.8%), this difference approaching but not reaching statistical significance (*p* = 0.087). However, although the overall frequency of recurrence was higher in our study, Holmstrup et al. reported a higher rate of MT (11/94, 11.7%), which, interestingly, occurred in 4 out of 12 recurring lesions in their series [27].

In the study by Sundberg et al. [12], out of 103 patients with surgically excised OLs, 43 (41.8%) developed recurrence, including 23/41 (56.1%) patients with non-homogeneous OLs and 20/62 (32.3%) patients with homogeneous OLs. These findings are in accord with our data, supporting the high incidence of recurrence in OL, despite adequate removal, and further substantiating an increased risk of recurrence for non-homogeneous lesions. Again, a relationship between recurrence and risk of MT was shown by Sundberg et al., as all four patients developing OSCC belonged to the group with recurring OL [12].

Kuribayashi et al. [19] reported a recurrence rate of 15.1% (8/53 surgically removed OL lesions). Similar to our study, only one of their patients developed OSCC (1.9%). However, in contrast with our findings, as well as those of Holmstrup et al. [27] and Sundberg et al. [12], homogeneous OLs showed a higher recurrence rate compared to non-homogeneous. The recurrence rate has also been associated with the treatment method used; for example, Ishii et al. [28] showed that recurrences occurred in about 29% and 25% of OLs treated by laser surgery and surgical excision, respectively, while Monteiro et al. [29] compared different surgical modalities (including scalpel excision vs. different types of laser) and did not find differences in the recurrence rate. In a recent systematic review [30], the authors concluded that surgical laser excision may lower OL recurrence rate compared to conventional treatment, although it does not affect the risk of MT. Such discrepancies among the aforementioned studies may be due to sample bias and methodological differences, such as variations in study design, management and screening protocols. In addition, it should be noted that, independent of the surgical method used to completely remove a lesion within clinically or even microscopically healthy margins, there is always the possibility that molecular changes may persist in the surrounding tissues, possibly giving rise to recurrences; in this regard, recent publications have highlighted the existence of molecular changes in surgical margins of OL and have suggested that the use of non-invasive diagnostic adjuncts, such as autofluorescence, may enhance visualization of actual borders of the lesion, possibly facilitating complete excision in clear molecular margins [15,31,32].

Besides homogeneity vs. non-homogeneity, other clinical characteristics of OL have also been reported to affect its biologic behavior. It is well established that increased size (>200 mm^2^) and site of involvement (tongue and floor of mouth) show significant association with cancer development [13]. Inconclusive data can be drawn from previous studies regarding the effect of these parameters in recurrence. Kuribayashi et al. [19] reported a positive correlation between recurrence and involvement of the gingiva, while Chainani-Wu et al. [33] demonstrated that early recurrence of OLs removed by CO_2_ laser surgery was significantly associated only with poor accessibility of the lesion margins, such as gingival lesions with facial and palatal involvement or extensive lesions in posterior locations. On the other hand, other studies have failed to show an association between lesion location and recurrence [12,28]. Our results did not disclose significant associations of local recurrence with the site of OL, although lesions located in palatal mucosa appeared to recur more frequently (66.7%), compared to gingiva and alveolar mucosa (34.3%), ventral tongue/floor of mouth (33.3%), buccal mucosa (29.0%) and lateral/dorsal tongue (15.4%). Similarly, size did not significantly correlate with risk of recurrence, despite that fact that 40.7% of larger (≥2.0 cm) PLs recurred compared to 29.0% of smaller (<2.0 cm) PLs.

In addition to clinical features, diverse demographic and social characteristics of OL patients have been extensively investigated regarding their risk of cancer development. More precisely, females and non-smokers are considered to display a higher potential for MT [13] and this socio-demographic profile has been suggested as a diagnostic criterion for proliferative verrucous leukoplakia (PVL), which is by definition a more aggressive entity [34]. Additionally, increased age is a risk factor for OSCC occurrence in OL patients [13]. The aforementioned literature on the risk of recurrence failed to disclose similar age-related associations [12,19,33] with the exception of Kuribayashi et al. [19], who reported a higher rate of recurrence in older patients (≥59 years). Further, neither gender nor smoking and drinking habits showed correlation with recurrence, similar to our findings.

It is widely acknowledged that oral epithelial dysplasia remains the most crucial predictor of OL progression, as most studies have shown that higher grades of dysplasia (such as moderately and severely dysplastic lesions, or high-grade lesions upon the binary grading system) carry an increased risk of cancer development compared to non-dysplastic or mildly dysplastic (or low grade) lesions [6,13,14,21,35]. For example, Warnakulashuriya et al. [36] have reported MT rates of 4.8%, 15.7% and 26.7% for mild, moderate and severe dysplasia, respectively, supporting that the severity of dysplasia bears significance as predictor for cancer development, while Kujan et al. [35], using the binary system, demonstrated a much higher rate of MT for high-grade vs. low-grade lesions (80% vs. 15%). This has led to individualized management protocols based on the degree of dysplasia, with several authors supporting a lenient follow-up without excision for hyperplastic lesions or even certain mild dysplasias of low suspicion [37]. Despite the unquestionable correlation between risk of MT and presence and degree of dysplasia, which, however, by itself cannot be considered as a reliable factor to predict the behavior of a given lesion, the association of dysplasia with increased likelihood of recurrences remains ambiguous. More precisely, previous studies did not display significant associations between dysplasia and OL recurrence [12,19,28], similarly to our study, in which, however, dysplastic lesions recurred at a higher percentage compared to hyperplastic lesions (34.5% vs. 15.4%). These findings could strengthen the notion of a more interventional approach in the management of all OLs (i.e., complete removal and close follow-up) independently of the degree of dysplasia. Recent molecular findings seem to further support the behavioral similarities between dysplastic and non-dysplastic OLs, in that hyperkeratotic OLs with no dysplasia display similar molecular characteristics with dysplastic OLs [38].

Further to sociodemographic, clinical and histopathologic features, we additionally investigated selected biomarkers that are deregulated during cancer. More specifically, we assessed the immunohistochemical expression of molecules that are involved in the signal transduction and activator of transcription 3 (STAT3) oncogenic signaling pathway by exerting proliferative and/or antiapoptotic effects [20,39]. In terms of oral carcinogenesis, these biomarkers have previously been investigated to a variable extent both in OSCC and OPMDs [14]; however, their correlation with risk of OL recurrence remains unknown.

STAT3 has been characterized as an oncogenic molecule, which is activated by different upstream events, conveys messages to the nucleus and drives the transcription of molecules promoting cell proliferation (such as cyclin D1 and other regulators of the cell cycle) [40] and inhibiting apoptosis (such as Bcl-xL and survivin) [20,41]. STAT3 aberrant expression has been associated with poor clinical outcome in OSCC [42,43,44]. In addition, it has been demonstrated that dysplastic lesions are characterized by increased STAT3 expression levels [43]. Nevertheless, in our study, tyrosine phosphorylated (activated) STAT3, despite its expression in the majority of OLs, did not show a correlation with degree of dysplasia nor was a predictor of recurrence.

Among STAT3 downstream molecules, cyclin D1 has also been implicated in the biologic behavior of OSCC [45]. Concerning oral premalignancy, an association of cyclin D1 expression with progression of the epithelium to a more dysplastic stage has been reported [46]; however, the potential prognostic value of cyclin D1 in terms of OL recurrence and/or MT is uncertain. In our study, a correlation between cyclin D1 expression and degree of dysplasia was noticed; however, no association with recurrent behavior was seen. Similarly, Ki-67 index, a major indicator of the cell proliferative activity, was found to be positively associated with the degree of dysplasia, in accord with previous publications [47]. Nonetheless, we did not find any correlation between Ki-67 levels and risk of recurrence, despite the fact that this molecule has been previously associated with progression of OL lesions [48,49,50].

In addition to cell proliferation, the STAT3 signaling pathway also regulates the expression of downstream molecules that exert antiapoptotic activity. Specifically, Bcl-xL is induced by oncogenic STAT3 signaling and displays elevated expression in various types of cancer, including head and neck SCC [51,52]. Further, overexpression of Bcl-xL in OSCCs is related to advanced tumor stages, locoregional lymph node metastasis, and degree of differentiation [53,54]. However, there is very limited available information on the expression of Bcl-xL in oral premalignancy; Schoelch et al. [55] reported that Bcl-X (including both splice variants Bcl-xL and Bcl-xS) demonstrated immunohistochemical positivity in 70% of hyperkeratotic and mildly dysplastic lesions and 85.7% of moderate/severe dysplasias and carcinomas in situ, concluding that this molecule is expressed early in the process of oral carcinogenesis. In our study, Bcl-xL was expressed in the majority of both primary and recurrent lesions (82.4% and 94.7%, respectively), not being associated with the presence and degree of dysplasia. Interestingly, comparison between non-recurrent and recurrent PLs revealed lower Bcl-xL expression levels in the latter group; indeed, lower Bcl-xL percentage, intensity and total IHC scores were identified as significant risk factors for OL recurrence in univariate analysis, the percentage score also remaining significant in multivariate analysis.

Survivin, another downstream molecule of the STAT3 signaling pathway and a well-known inhibitor of apoptosis, has been shown to be expressed in oral premalignancy [56] displaying positive correlations with dysplastic phenotype [57] and progression to malignancy [58]. However, no study to this date has investigated the relationship between the expression of this marker and the risk of OL recurrence. Our findings did not support an association between survivin IHC expression and degree of dysplasia. However, surprisingly, its expression correlated inversely with the risk of local recurrence with decreased levels of survivin in recurrent cases; in univariate (but not multivariate) analysis, survivin percentage score emerged as a significant risk factor for recurrence. These data highlight the complexity of oral carcinogenesis and suggest the possibility that various molecules may be involved in different and sometimes unexpected ways in diverse aspects of progression, i.e., recurrence vs. MT. Nonetheless, validation and clarification of these findings and the potential role of specific molecules as predictors of recurrence necessitate larger studies investigating these immunomarkers in recurrent vs. non-recurrent OLs.

Another investigation carried out in our study was the comparison between PLs and their corresponding recurrences. Understanding how the clinical, histopathologic, and IHC features of OL lesions evolve through time and differ between first occurrence and recurrence is important. First of all, characterization of the biologic behavior of OPMDs as a function of time may give insight into the mechanisms of persistence and recurrence, possibly providing useful clues for the proper management of these lesions. Interestingly, we noticed a significantly milder clinicopathologic phenotype in the recurrent lesions, which tended to be significantly smaller and more homogeneous, also including a higher percentage of hyperplastic (non-dysplastic) cases. This should not be interpreted as an indication of a better biologic behavior in recurrences compared to PLs, but probably implies that proper follow-up could result into early detection of these lesions before they acquire aggressive phenotypic and molecular characteristics, thus highlighting the necessity of close monitoring of these patients. On the other hand, it is noteworthy that recurrences showed significantly higher expression levels of pSTAT3 and Bcl-xL (and a tendency also for higher expression of cyclin D1 and survivin) suggesting that, despite the lack of clinicopathologic features of aggressiveness, it is likely that recurrences may be characterized by an increased level of molecular aberrations, potentially linked to their risk of further progression.

## 5. Conclusions

We attempted to characterize the sociodemographic, clinical, microscopic and certain IHC features in relation to OL recurrence. Despite the relatively small size of our patient cohort, our approach analyzing all individual lesions of each patient and categorizing them as primary lesions (at first visit or during follow-up) and recurrences, allowed us to investigate parameters associated with recurrence. Based on our data, these recurrence-related parameters may differ to a large extent from those that have been described as affecting the risk of progression to malignancy. We also showed that altered expression of specific molecules could herald recurrence, which, if validated by future studies, could result in the application of predictors of recurrence in the management of these patients. Finally, we demonstrated that recurrent OLs may show a less aggressive clinicopathologic phenotype compared to their primary counterparts, possibly due to early detection during follow-up, although the overexpression of specific oncogenic molecules may be linked to their occurrence and/or risk of further progression. Collectively, our findings should be considered as supportive of the notion that, until more reliable predictors of progression are available, current OL management protocols should favor excision of all histopathologically proven lesions and close monitoring. These important clinical notions need to be further assessed in larger studies involving more patients followed up for a lengthy period of time.

## Figures and Tables

**Table 1:** Frequency distribution of demographics, tobacco and alcohol habits, and clinical and histopathologic parameters of 135 oral leukoplakia (OL) lesions, including primary lesions and recurrences.

Parameters		All Lesions		Primaries		Recurrences	
		*n*	(%)	*n*	(%)	*n*	(%)
Number of lesions (*n*)		135	(100%)	97	(71.9%)	38	(28.1%)
Demographics							
	Gender						
	Male	58	(43%)	42	(43.3%)	16	(42.1%)
	Female	77	(57%)	55	(56.7%)	22	(57.9%)
	Age (years)						
	>50	101	(74.8%)	72	(74.2%)	29	(76.3%)
	≤50	34	(25.2%)	25	(25.8%)	9	(23.7%)
Patient habits							
	Tobacco habits						
	Use (Smoker)	66	(48.9%)	53	(55.6%)	13	(34.2%)
	Non-use (Ex-smoker/ Non-smoker)	69	(51.1%)	44	(45.4%)	25	(65.8%)
	Alcohol consumption						
	Daily drinker	53	(39.3%)	40	(41.2%)	13	(34.2%)
	Non-drinker/Social drinker	82	(60.7%)	57	(58.8%)	25	(65.8%)
Clinical parameters							
	Site						
	Buccal mucosa	44	(32.6%)	32	(33.0%)	12	(31.6%)
	Ventral tongue–floor of mouth	17	(12.6%)	11	(11.3%)	6	(15.8%)
	Dorsal and lateral tongue	15	(11.1%)	13	(13.4%)	2	(5.3%)
	Mandibular gingiva and alveolus	31	(23%)	22	(22.7%)	9	(23.7%)
	Maxillary gingiva and alveolus	18	(13.3%)	13	(13.4%)	5	(13.2%)
	Hard palate	10	(7.4%)	6	(6.2%)	4	(10.5%)
	Largest dimension (cm)						
	≥2.0	28	(20.7%)	27	(27.8%)	1	(2.6%)
	<2.0	103	(76.3%)	69	(71.1%)	34	(89.5%)
	Missing data	4	(3.0%)	1	(1.0%)	3	(7.9%)
	Homogeneity						
	Non-homogenous	32	(23.7%)	26	(26.8%)	6	(15.8%)
	Homogenous	103	(76.3%)	71	(73.2%)	32	(84.2%)
	Treatment method						
	LASER	65	(48.1%)	52	(53.6%)	13	(34.2%)
	Surgical excision	70	(51.9%)	45	(46.4%)	25	(65.8%)
Histopathologic diagnosis							
	Severe dysplasia	3	(2.2%)	2	(2.1%)	1	(2.6%)
	Moderate dysplasia	28	(20.7%)	21	(21.6%)	7	(18.4%)
	Mild dysplasia	80	(59.3%)	61	(62.9%)	19	(50.0%)
	Hyperplasia	24	(17.8%)	13	(13.4%)	11	(28.9%)

**Table 2:** Frequency distribution and comparison of demographics, tobacco and alcohol habits, and clinical and histopathologic parameters between recurrent and non-recurrent primary oral leukoplakia (OL) lesions.

Parameters		Primary OL Lesions									
		Recurrent									
		Yes			No			*p* *		Total	
		*n*	(%)		*n*	(%)				*n*	(%)
Number of lesions		31	(32.0%)		66	(68.0%)				97	(100%)
Demographic data											
	Gender										
	Male	12	(38.7%)		30	(45.5%)		0.661		42	(43.3%)
	Female	19	(61.3%)		36	(54.5%)				55	(56.7%)
	Age (years)										
	>50	23	(74.2%)		49	(74.2%)		1.000		72	(74.2%)
	≤50	8	(25.8%)		17	(25.8%)				25	(25.8%)
Patient’s habits											
	Tobacco habits										
	Use (Smoker)	18	(58.1%)		35	(53.0%)		0.668		53	(54.6%)
	Non-use (Ex-smoker/Non-smoker)	13	(41.9%)		31	(47.0%)				44	(45.4%)
	Alcohol consumption										
	Daily drinker	3	(9.7%)		17	(25.8%)		0.105		20	(20.6%)
	Non-drinker/Social drinker	28	(90.3%)		49	(74.2%)				77	(79.4%)
Clinical parameters											
	Site										
	Buccal mucosa	9	(29.0%)		22	(33.3%)		0.405		31	(32.0%)
	Ventral tongue-Floor of mouth	4	(12.9%)		8	(12.1%)				12	(12.4%)
	Dorsum and lateral tongue	2	(6.5%)		11	(16.7%)				13	(13.4%)
	Mandibular gingival and alveolar	7	(22.6%)		15	(22.7%)				22	(22.7%)
	Maxillary gingival and alveolar	5	(16.1%)		8	(12.1%)				13	(13.4%)
	Hard Palate	4	(12.9%)		2	(3.0%)				6	(6.2%)
	Largest dimension (cm)										
	≥2.0	11	(35.5%)		16	(24.2%)		0.333		27	(27.8%)
	<2.0	20	(64.5%)		49	(74.2%)				69	(71.1%)
	Missing data	0	(0%)		1	(1.5%)				1	(1.0%)
	Homogeneity										
	Non-homogenous	12	(38.7%)		14	(21.2%)		0.087		26	(26.8%)
	Homogenous	19	(61.3%)		52	(78.8%)				71	(73.2%)
	Treatment method										
	LASER	19	(61.3%)		33	(50.0%)		0.383		52	(53.6%)
	Surgical excision	12	(38.7%)		33	(50.0%)				45	(46.4%)
Histopathologic diagnosis											
	Severe dysplasia	0	(0%)		2	(3.0%)		0.237		2	(2.1%)
	Moderate dysplasia	5	(16.1%)		16	(24.2%)				21	(21.6%)
	Mild dysplasia	24	(77.4%)		37	(56.1%)				61	(62.9%)
	Hyperplasia	2	(6.5%)		11	(16.7%)				13	(13.4%)
	Surgical excision margins										
	Positive	5	(16.1%)		14	(21.2%)		1.000		19	(19.6%)
	Negative	7	(22.6%)		19	(28.8%)				26	(26.8%)

* Two-tailed Fisher’s exact test.

**Table 3:** Synopsis of immunohistochemical results for the various studied molecules in oral leukoplakia (OL) lesions, including primary lesions and recurrences.

Parameters	All Lesions		Primaries		Recurrences	
**Cyclin D1**						
No. of positive cases (%)	101/104	(97.1%)	82/85	(96.5%)	19/19	(100%)
Percentage cell score (mean and range)	1.00	(0–2)	0.97	(0–2)	1.0	(1–1)
Intensity score (mean and range)	1.50	(0–3)	1.44	(0–3)	1.84	(1–3)
Total score (mean and range)	2.50	(0–4)	2.41	(0–4)	2.84	(2-4)
pSTAT3						
No. of positive cases (%)	67/104	(64.4%)	52/85	(61.2%)	15/19	(78.9%)
Percentage cell score (mean and range)	0.90	(0–3)	0.84	(0–3)	1.26	(0–3)
Intensity score (mean and range)	1.00	(0–3)	0.88	(0–3)	1.63	(0–3)
Total score (mean and range)	1.90	(0–6)	1.72	(0–6)	2.89	(0–6)
BCL-xL						
No. of positive cases (%)	88/104	(84.6%)	70/85	(82.4%)	18/19	(94.7%)
Percentage cell score (mean and range)	1.50	(0–3)	1.47	(0–3)	1.79	(0–3)
Intensity score (mean and range)	1.40	(0–3)	1.29	(0–3)	1.79	(0–3)
Total score (mean and range)	2.90	(0–6)	2.76	(0–6)	3.58	(0–6)
Survivin						
No. of positive cases (%)	100/104	(96.2%)	84/85	(98.8%)	16/19	(84.2%)
Percentage cell score (mean and range)	2.10	(0–3)	2.17	(0–3)	1.89	(0–3)
Intensity score (mean and range)	2.20	(0–3)	2.26	(0–3)	1.84	(0–3)
Total score (mean and range)	4.30	(0–6)	4.43	(0–6)	3.74	(0–6)
Ki-67						
No. of positive cases (%)	104/104	(100%)	85/85	(100%)	19/19	(100%)
Percentage cell score (mean and range)	1.10	(1–2)	1.14	(1–2)	1.11	(1–2)

**Table 4:** Comparison of immunohistochemical results of various studied molecules between recurrent and non-recurrent primary oral leukoplakia (PLs) lesions.

	Primary OL Lesions		
	Recurrent		
	Yes	No	*p* *
Immunohistochemical Scores	Median (Range)	Median (Range)	
**Cyclin D1**			
Percentage cell	1.0 (0–1)	1.0 (0–2)	0.271
Intensity	1.0 (0–3)	1.0 (0–3)	0.914
Total	2.0 (0–4)	2.0 (0–4)	0.735
pSTAT3			
Percentage cell	1.0 (0–2)	1.0 (0–3)	0.589
Intensity	1.0 (0–2)	1.0 (0–3)	0.361
Total	2.0 (0–4)	2.0 (0–6)	0.315
Bcl-xL			
Percentage cell	1.0 (0–3)	2.0 (0–3)	<0.001
Intensity	1.0 (0–2)	1.0 (0–3)	<0.001
Total	2.0 (0–5)	3.0 (0–6)	<0.001
Survivin			
Percentage cell	1.0 (1–3)	3.0 (0–3)	<0.001
Intensity	2.0 (1–3)	2.0 (0–3)	0.372
Total	3.0 (2–6)	5.0 (0–6)	0.006
Ki-67			
Total	1.0 (1–2)	1.0 (1–2)	0.213

* Two-tailed Fisher’s exact test.

**Table 5:** Uni-variable Cox regression analysis of risk factors for the recurrence of primary oral leukoplakia (PLs) lesions.

Risk Factors		Cox Regression Uni-Variable Analysis		
		HR	(95% CI)	*p*
Demographics				
	Gender			
	Male (Ref: Female)	0.762	(0.370–1.572)	0.462
	Age (years)			
	>50 years of age (Ref: ≤50 years of age)	1.695	(0.746–3.852)	0.207
Patient habits				
	Tobacco habits			
	Use (Ref: Non-use)	0.974	(0.477–1.992)	0.944
	Alcohol consumption			
	Use (Ref: No alcohol intake)	0.523	(0.248–1.102)	0.088
Clinical parameters				
	Subsite			
	Buccal mucosa (Ref: All other sites)	0.890	(0.409–1.939)	0.770
	Ventral tongue–floor of mouth (Ref: All other sites)	0.948	(0.331–2.716)	0.921
	Dorsal and lateral tongue (Ref: All other sites)	0.554	(0.132–2.326)	0.420
	Mandibular gingiva and alveolus (Ref: All other sites)	0.937	(0.403–2.178)	0.880
	Maxillary gingiva and alveolus (Ref: All other sites)	1.539	(0.590–4.016)	0.379
	Hard palate (Ref: All other sites)	1.483	(0.515–4.265)	0.465
	Largest dimension (cm)			
	≥2cm (Ref: <2.0 cm)	1.225	(0.586–2562)	0.589
	Homogeneity			
	Non-Homogenous (Ref: Homogenous)	1.735	(0.840–3.582)	0.136
	Treatment method			
	LASER (Ref: Surgical excision)	1.228	(0.595–2.538)	0.579
Histopathologic parameters				
	Diagnosis (From hyperplasia to severe dysplasia)	0.975	(0.571–1.666)	0.927
	Excision margins (for surgical excision only)			
	Positive (Ref: Negative)	1.043	(0.329–3.305)	0.944
IHC scores				
Cyclin D1				
	Percentage of cells score	0.467	(0.128–1.706)	0.250
	Intensity score	0.920	(0.527–1.609)	0.771
	Total score	0.872	(0.552–1.378)	0.557
pSTAT-3				
	Percentage of cells score	0.690	(0.403–1.183)	0.177
	Intensity score	0.674	(0.403–1.126)	0.132
	Total score	0.817	(0.625–1.068)	0.140
BCL-xL				
	Percentage of cells score	0.442	(0.271–0.720)	0.001
	Intensity score	0.404	(0.230–0.711)	0.002
	Total score	0.644	(0.496–0.836)	0.001
Survivin				
	Percentage of cells score	0.511	(0.329–0.792)	0.003
	Intensity score	0.972	(0.600–1.575)	0.909
	Total score	0.806	(0.634–1.023)	0.077
Ki-67				
	Total score	1.743	(0.653–4.656)	0.268

**Table 6:** Comparison of clinical (size and homogeneity) and histopathologic parameters between primary oral leukoplakia lesions (PLs) that recurred and their corresponding first recurrences.

Parameters		PLs and Corresponding Recurrences					
		PLs (Recurrent)			1st Recurrence		*p* *
		*n*	(%)		*n*	(%)	
Number of lesions		31			31		
Clinical parameters							
	Size (cm)						
	≥2.0	11	(35.5%)		0	(0%)	<0.001
	<2.0	20	(64.5%)		31	(100%)	
	Homogeneity						
	Non-homogenous	12	(38.7%)		4	(12.9%)	0.040
	Homogenous	19	(61.3%)		27	(87.1%)	
Histopathologic diagnosis							
	Hyperplasia	2	(6.5%)		10	(32.3%)	0.028
	Mild dysplasia	24	(77.4%)		15	(48.4%)	
	Moderate dysplasia	5	(16.1%)		5	(16.1%)	
	Severe dysplasia	0	(0%)		1	(3.2%)	

* Two-tailed Fisher’s exact test.

**Table 7:** Comparison of immunohistochemical results of various studied molecules between primary oral leukoplakia lesions (PLs) that recurred and their corresponding first recurrences.

PLS and Corresponding Recurrences							
Parameters		PLs (Recurrent)			1st Recurrence		*p*
Cyclin D1							
Percentage of cells score (median and range)		1.0	(0–1)		1.0	(1–1)	0.157
Intensity score (median and range)		1.0	(0–3)		1.0	(1–3)	0.122
Total score (median and range)		2.0	(0–4)		2.0	(2–4)	0.070
pSTAT3							
Percentage of cells score (median and range)		1.0	(0–2)		1.0	(0–3)	0.054
Intensity score (median and range)		1.0	(0–2)		2.0	(0–3)	0.009
Total score (median and range)		2.0	(0–4)		3.0	(0–6)	0.018
Bcl-xL							
Percentage of cells score (median and range)		1.0	(0–3)		2.0	(0–3)	0.007
Intensity score (median and range)		1.0	(0–2)		2.0	(0–3)	0.006
Total score (median and range)		2.0	(0V5)		4.0	(0–6)	0.005
Survivin							
Percentage of cells score (median and range)		1.0	(1–3)		2.0	(0–3)	0.541
Intensity score (median and range)		2.0	(1–3)		2.0	(0–3)	0.059
Total score (median and range)		3.0	(2–6)		4.0	(0–6)	0.645
Ki-67							
Total score (median and range)		1.0	(1–2)		1.0	(1–2)	0.564

## Data Availability

Data available on request due to restrictions.

## Abbreviations

OL: Oral leukoplakia; MT: malignant transformation; PLs: primary lesions; OSCC: oral squamous cell carcinoma; OPMD: oral potentially malignant disorder; WHO: World Health Organization; IHC: immunohistochemical; NKUA: National and Kapodistrian University of Athens; H&E: hematoxylin and eosin-stained; DFS: Disease free survival; PLFV: Primary lesions noticed at first visit; PLFU: Primary lesions noticed at follow-up; RFV: Recurrences of primary lesions noticed at first visit; RFU: Recurrences of primary lesions noticed at follow-up; (*n*): Number of lesions.
